# Testing a Liquid Crystal Visual Thermometer Device in Newborns and Young Infants

**DOI:** 10.7759/cureus.84802

**Published:** 2025-05-25

**Authors:** Bolanle Aishat Kasali, Jennifer Udeogu, Anna Strauss, Jack M Wolf, Ann M Brearley, Iretiola B Fajolu, Rashedat Oshodi, Abigail Obi, Morgan McBride, Sanjana Molleti, Tina M Slusher, Chinyere Ezeaka, Ifelayo Ojo

**Affiliations:** 1 Department of Pediatrics, University of Minnesota, Minneapolis, USA; 2 Department of Pediatrics, University of Minnesota Medical School, Minneapolis, USA; 3 School of Public Health, University of Minnesota, Minneapolis, USA; 4 Department of Pediatrics, Lagos University Teaching Hospital (LUTH), Lagos, NGA; 5 College of Medicine, University of Lagos, Lagos, NGA; 6 Department of Pediatrics, University of Minnesota Masonic Children's Hospital (UMMCH), Minneapolis, USA; 7 Department of Pediatrics, Hennepin County Medical Center, Minneapolis, USA

**Keywords:** filtered sunlight phototherapy, liquid crystal thermometer, neonatal hyperthermia, neonatal hypothermia, neonatal jaundice, temperature monitoring

## Abstract

Objective

Severe neonatal jaundice is a major cause of death and disability among newborns in low- and middle-income countries (LMICs). Filtered sunlight phototherapy (FSPT) is safe and effective but requires hourly temperature monitoring to detect hyperthermia/hypothermia. This need for close temperature monitoring by family members and community health extension workers is impeding FSPT scale-up in LMICs, where healthcare providers are scarce and nurseries are understaffed. The ability of a caregiver to accurately measure temperature can affect infant health outcomes in many other illnesses as well. This study aims to evaluate the accuracy and usability of a modified Liquid Crystal Thermometer Device (LCTD) strip, modified from the original ThermoSpot™ (Maternova, Providence, RI), for caregivers and healthcare providers in measuring infant temperatures. The modification was attempted to improve monitoring for hyperthermia better than the original ThermoSpot™, which had only one color for hyperthermia but three colors for hypothermia, and changed as the infants got colder.

Methods

We conducted a cross-sectional study of infants (zero to six months) at the University of Minnesota Masonic Children’s Hospital (UMMCH) in Minnesota and Lagos University Teaching Hospital (LUTH) in Nigeria. Analysis was limited to 40 infants enrolled at LUTH following preliminary data review. We compared standard of care (SoC) temperatures using digital thermometers with LCTD readings at five different time points over two hours. Photos of the LCTD strips were taken at each time point and were later reviewed by researchers.

Results

Forty infants were studied to determine the agreement between LCTD readings and SoC temperatures. These results were then compared between caregivers and researchers. The average agreement between all readers (caregivers and research assistants) was 64%. After a methodological adjustment to remove the initial LCTD temperature reading from each infant’s dataset, the overall agreement rate between caregivers and research assistants was 63.5%. We also separately analyzed the accuracy of caregiver and research assistant readings of the LCTD strip when compared to the SoC temperature, finding that caregivers had an overall accuracy of 65%, while the research assistants had an overall accuracy of 63%. Lastly, we conducted a blinded photo analysis among research assistants to determine whether LCTD readings were being interpreted consistently between researchers, and found a slight bias for higher temperatures: for readings at or above 37°C, the interpreted temperature tended to be higher than the measured temperature.

Conclusion

The modified LCTD did not correlate well with digital thermometer temperature readings. Further modifications are needed to make it useful for managing neonates under FSPT and other conditions/illnesses where temperature monitoring is advised. There remains a gap in providing illiterate caregivers a reliable way to determine if their infant is normothermic and safe under FSPT.

## Introduction

Severe neonatal jaundice (SNJ) poses a significant threat to the well-being of newborns in low- and middle-income countries (LMICs), contributing to elevated rates of mortality and disability. Within a cohort of neonates with jaundice reviewed by systemic review and meta-analysis by Diala et al., the prevalence of SNJ ranges from 8.31% to 31.49%, with the highest incidence observed in the African region [1]. Neonatal jaundice can be effectively managed with phototherapy. However, the limited availability, need for electricity, and high cost of standard phototherapy units hinder the ability of healthcare facilities in LMICs to procure them [2]. This highlights the need for more accessible and affordable phototherapy options in LMICs to effectively treat neonatal jaundice [2,3].

Filtered sunlight phototherapy (FSPT) has been tested and shown to be safe, efficacious, and a viable alternative to electricity-powered phototherapy for the treatment of jaundice [4,5]. However, FSPT currently requires at least hourly temperature monitoring by healthcare providers (HCPs) because infants receiving FSPT are prone to both hypothermia and hyperthermia [4,5]. This need for close temperature monitoring, especially in tropical climates affected by the ongoing impacts of global warming, is impeding FSPT scale-up in LMICs. Additionally, caregivers and HCPs often have difficulty measuring temperatures due to the unavailability of or difficulty reading thermometers, as well as understaffed nurseries [3,5]. This leads to suboptimal temperature management and points to an urgent need for a temperature monitoring device that is simple to use and able to be interpreted by an illiterate caregiver. Other conditions in infants also require close monitoring for both hypothermia and hyperthermia, including sick neonates, febrile illnesses (especially in those at risk for febrile seizures), meningoencephalitis, and traumatic brain injury. In LMICs, much of that monitoring may fall on family members or healthcare workers with limited training due to staff shortages.

The original ThermoSpot™ (Maternova, Providence, RI), a low-cost, easy-to-use and reuse Liquid Crystal Thermometer Device (LCTD), was developed with a focus on allowing mothers of premature infants to be able to monitor their own infant’s temperature and adjust care accordingly if their infant was becoming cold. It has worked well for this purpose [6-11]. On the original ThermoSpot™, light green represents “getting cold,” red represents “cold,” and black represents “extremely cold.” There is only one color (blue) for “hot.” The original ThermoSpot™ was tested in a small pilot study in Nigeria under FSPT, and has been presented in one short report and two posters [12]. However, because of the difficulty in grading the degree of hyperthermia using the original ThermoSpot™, part of this study team (TMS, IPO) approached Maternova and asked if they could develop an LCTD that could better grade hyperthermia than the original did. If an LCTD could be developed that graded hyperthermia in smaller increments, it would potentially allow mothers and HCPs to intervene earlier when an infant under FSPT was becoming warm, and before they got too hot. Possible interventions include putting an infant on a wet white towel while still under FSPT. This paper introduces the modified LCTD as a mode of monitoring temperature in infants. This strip was modified from the original ThermoSpot™.

This study aimed to assess the accuracy and usability of LCTD strips in measuring infants' body temperature to offer a simple and effective solution to the need for frequent temperature measurement and to minimize the burden on HCPs (primarily nurses). The research objectives involved evaluating the accuracy of LCTD strips in infants aged zero to six months and assessing the proficiency of caregivers and HCPs in interpreting LCTD readings. This was a collaborative study between the University of Minnesota (UMN) and the University of Minnesota Masonic Children’s Hospital (UMMCH) in the United States and the Lagos University Teaching Hospital (LUTH) in Nigeria.

This paper reports the results of accuracy comparisons between the LCTD strip and the standard digital thermometer across multiple time points. Additionally, the findings from a comparison of LCTD strip placement at different body locations (forehead vs. axilla vs. right upper quadrant (RUQ)) and their correlation with axillary body temperature, as measured using a digital thermometer, are discussed. The paper explores the application of this modified LCTD strip, shedding light on challenges encountered during the study and barriers to implementing the LCTD strip in LMICs. This research was previously presented at the Pediatric Academic Societies Meeting in May 2024 in Toronto, Canada (Poster: Kasali AB, Ojo IP, Udeogu J, Oshodi R, Slusher TM. Testing a Visual Thermometer in Newborns and Young Infants. Pediatric Academic Societies Meeting; May 2024).

## Materials and methods

Study design

We conducted a cross-sectional study of infants at UMMCH in Minnesota and LUTH in Nigeria. A sample size of 300 temperature measurements was estimated assuming a true device accuracy of 95%, and we would like to characterize that accuracy within a 3% margin of error at the 95% confidence level. Assuming that we would obtain three temperature measurements on each enrolled infant on average, we would need to enroll 100 infants. To account for missing data and for infants who would get less than the three measurements, we planned to enroll 120 infants. We compared standard of care (SoC) temperatures using digital thermometers with LCTD temperature readings obtained by research assistants and caregivers across five different time points.

Study setting and population

Participants were recruited from UMMCH in Minnesota and LUTH in Nigeria. Infants zero to six months old who were not undergoing active resuscitation were eligible. Consent was obtained from a parent or legal guardian who could consent in English. We excluded infants undergoing active resuscitation, any infant for whom the physician deemed study enrollment would interfere with their care, any infant whose parents declined enrollment, or infants with parents who did not speak English. Ethical approval was obtained from both UMN and LUTH IRBs.

Study device and procedure

**Figure 1:**
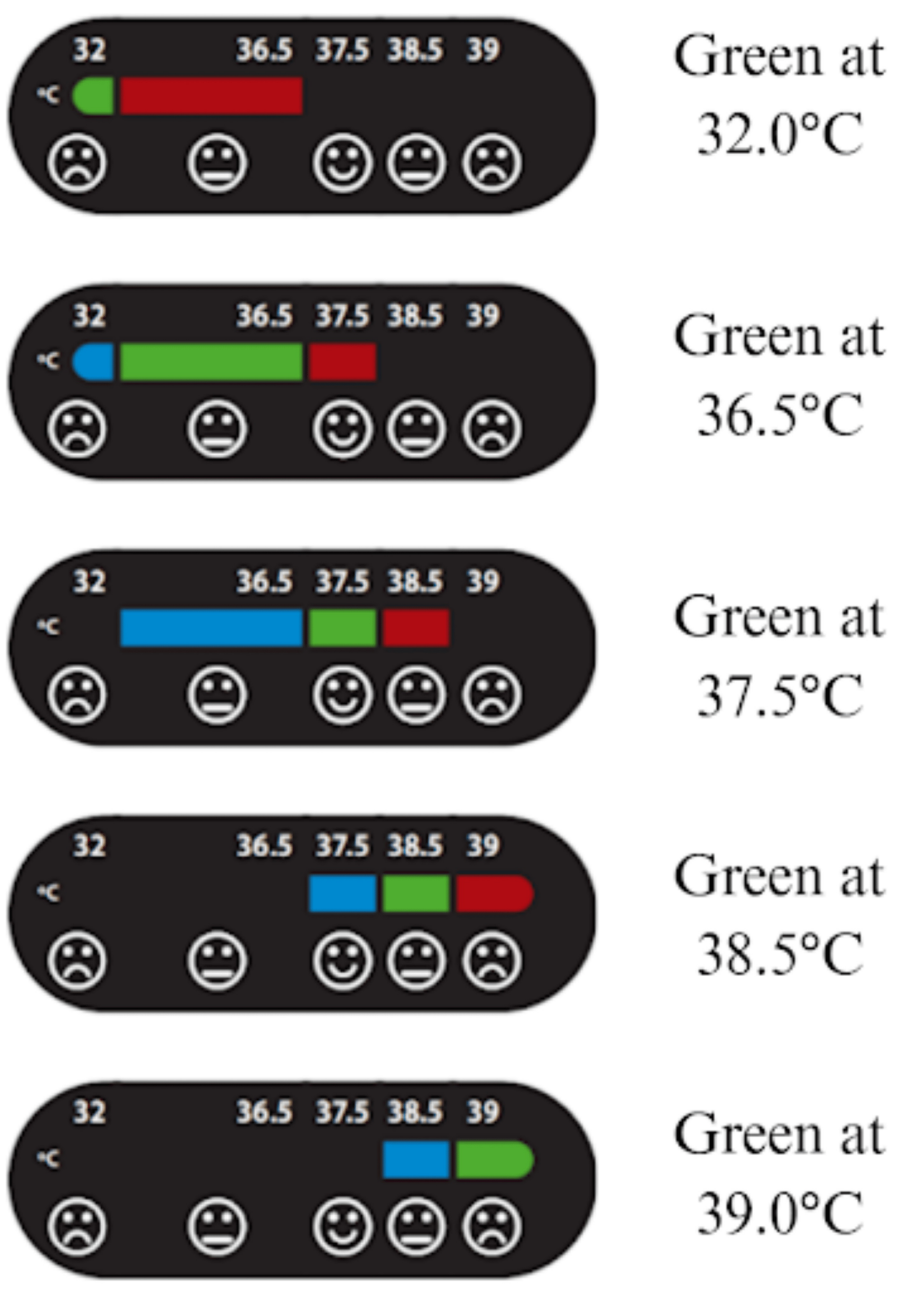
LCTD color gradient and interpretation of temperature This diagram depicts the LCTD strip color gradient, with green representing the accurate temperature reading per manufacturer instructions. Note that what is red in this figure was amber/light brown on observation. LCTD, Liquid Crystal Thermometer Device

The study used an LCTD strip that changes color at four distinct temperature cut-off points, giving five color ranges, including normal infant temperature (Figure 1). Developed by Maternova under our team’s direction, the oval-shaped LCTD strip (4 cm × 2 cm) consists of two layers of semiflexible dark plastic. The back has a peel-away lining like a sticker, while the front has windows revealing compartments of liquid crystals. Maternova provided instructions for reading the LCTD strip (Figure 1). The organization had no role in study design and execution.

The LCTD strip changes color based on body temperature, with five horizontal, box-shaped regions indicating temperature categories from “too cold” to “too hot” (i.e., “too cold,” “cold,” “normal,” “hot,” “too hot”). Placed on the infant’s temple, axilla, or RUQ of the abdomen, the device’s rightmost colored green box reflects the temperature. Cartoon faces beneath the boxes help users interpret the temperature: a smiley face for “normal,” neutral for “hot” or “cold,” and a frowny face for “too hot” or “too cold.” Research assistants were trained to use the strip and record temperatures. Both caregivers and research assistants observed and recorded the LCTD strip temperature display at five time points: initial reading (T0) within 15 minutes of the first SoC temperature, at 30 minutes after the first time point (T1), at 60 minutes (T2), at 90 minutes (T3), and 120 minutes (T4). These readings were later compared to standard thermometer temperature measurements taken at the same time points for accuracy.

We aimed to obtain the first LCTD strip temperature reading within 15 minutes of the SoC temperature recorded during triage (T0). After T0, the appropriate body sites were swabbed with an alcohol wipe, and an LCTD strip was applied. Researchers and caregivers interpreted the LCTD strip readings independently, and the researcher also took an axillary temperature at each time point for comparison. This process was repeated every 30 minutes for two hours, resulting in five readings per baby. The LCTD strips remained in place between readings. Data were stored in a REDCap database. Data collection ended if a baby was discharged or admitted before all readings were obtained.

**Figure 2:**
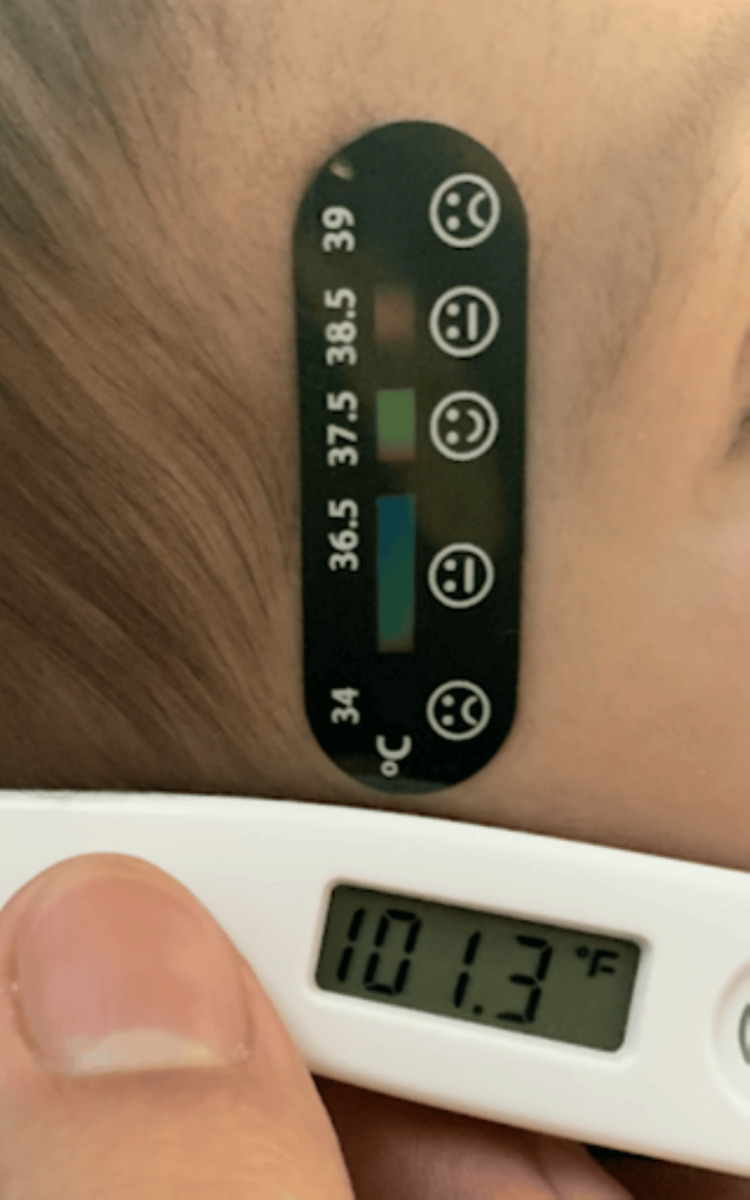
Comparison of LCTD strip reading with standard digital thermometer reading The LCTD strip at the temple displays "normal" temperature (smiley face), while the infant’s SoC temperature was more accurately measured as 101.3°F (38.5°C) with the standard digital thermometer. LCTD, Liquid Crystal Thermometer Device; SoC, standard of care

Results were analyzed for agreement between LCTD strip readings and measured SoC temperatures (Figure 2). The agreement rate was calculated as the number of LCTD strip readings that correctly matched the observed temperature divided by the total number of observations in each category. Accuracy was compared by body part location of the LCTD strip, by reader (caregiver vs. research assistant), and by temperature range. In addition, the effect of discarding the first reading on accuracy was assessed. Confidence intervals (CIs) for each agreement rate were calculated using generalized estimating equations to account for repeated measurements within infants. All data analysis was carried out using R statistical software version 4.4.1 (R Foundation for Statistical Computing, Vienna, Austria).

The study team analyzed preliminary data midway through the study and determined that there were inconsistencies in LCTD strip interpretation as well as discrepancies with color changes based on body part location (temple vs. axilla vs. RUQ). The manufacturer was contacted and cited the axilla as the most accurate body site for temperature reading. Updated instructions on how to read the LCTD strip were agreed upon and sent to all research assistants. All data collected after this modification occurred with the LCTD being placed on the axilla alone.

In addition, photos of the LCTD strips at the time of the temperature readings were taken and were reviewed later by research assistants. The research assistants were blinded to the actual recorded temperatures corresponding to each photo. They recorded readings into a separate spreadsheet, noting if the photo was uninterpretable or if two temperature ranges were both plausible. We calculated the percentage of times the research assistants indicated the photo could not be interpreted, and the percentage of times the research assistants listed two plausible temperature ranges.

## Results

Study enrollment started in December 2022 at the UMMCH site and closed in June 2023. A total of 23 infants were enrolled at UMMCH. Preliminary data analysis indicated that the LCTD readings were “too cold” for nearly all infants. In reality, only one infant’s SoC measured temperature was in the “too cold” range, indicating potential issues either with the device itself, the attachment of the device, or the protocol for reading the device. This study arm was dropped from further analysis.

Study enrollment started in March 2023 at the LUTH site. The study closed for enrollment in November 2023. A total of 97 infants were enrolled with a median age of three weeks (interquartile range (IQR): one to eight weeks). After an initial analysis in June 2023, the study team contacted Maternova, who recommended revising the study protocol to improve the accuracy of the readings. After the protocol revision, 40 infants were enrolled at the LUTH site with a median age of four weeks (IQR: two to 15 weeks).

Comparison of accuracy: LCTD vs. digital thermometer

**Figure 3:**
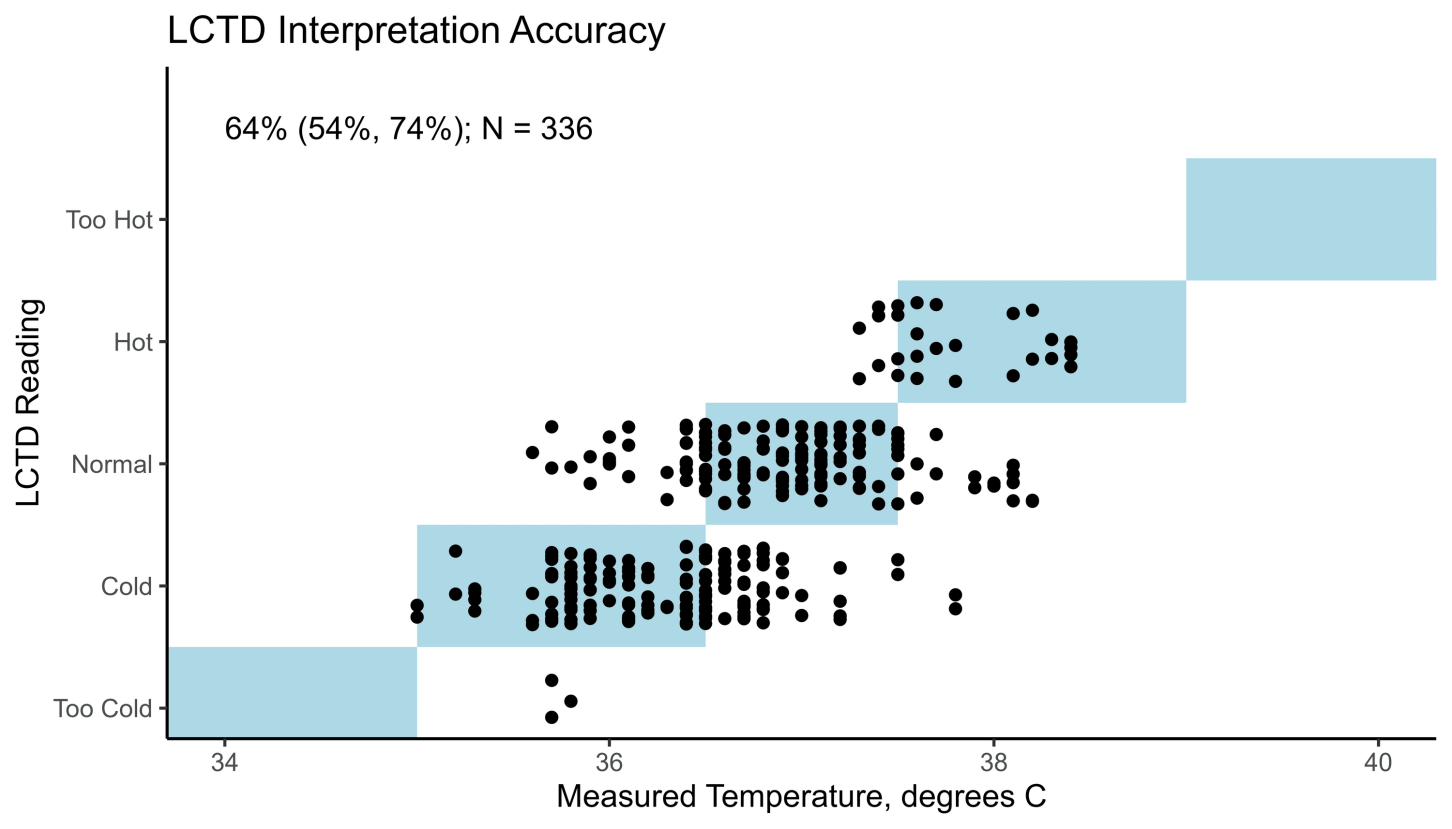
Comparison of LCTD readings by caregivers and research assistants (vertical axis) and measured SoC temperature (horizontal axis) Points represent 336 LCTD readings from both research assistants and caregivers corresponding to 168 temperature readings across 40 infants. Points within the light blue boxes on the diagonal indicate observations where the LCTD reading implied a temperature range containing the measured SoC temperature. The estimated accuracy (95% confidence interval) is reported in the upper left corner. Note that points have been jittered vertically for visual clarity. LCTD, Liquid Crystal Thermometer Device; SoC, standard of care

Analysis was limited to the 40 infants enrolled after the protocol revision. Among these 40 infants, 25 had five SoC temperature readings, six had four readings, eight had three readings, and one had two readings for a total of 175 SoC temperature readings (Table 1). No LCTD reading was obtained for seven of these SoC temperature readings, resulting in 168 LCTD observations. The agreement between the recorded SoC temperatures and the LCTD readings by the caregivers and the research assistants is shown in Figure 3. The agreement rates (95% CIs) for these 40 infants were 75% (57%, 93%) for “cold,” 59% (42%, 75%) for “normal,” 51% (15%, 87%) for “hot,” and 64% (54%, 74%) overall, implying a 64% accuracy of LCTD temperature readings when compared with recorded SoC temperatures.

**Table 1:** Study subject demographics This table shows demographics and baseline data for the 40 study subjects enrolled at Lagos University Teaching Hospital (LUTH) in Nigeria after the protocol change. IQR, interquartile range

Characteristic	N = 40 infants
Age in weeks, median (IQR)	4 (2, 15)
Older than 30 days, n (%)	20 (50%)
Completed SoC temperature measurements per infant, n (%)	
2	1 (2.5%)
3	8 (20%)
4	6 (15%)
5	25 (63%)
Temperature at initial measurement, °C, median (IQR)	36.5 (36.1, 36.9)

**Figure 4:**
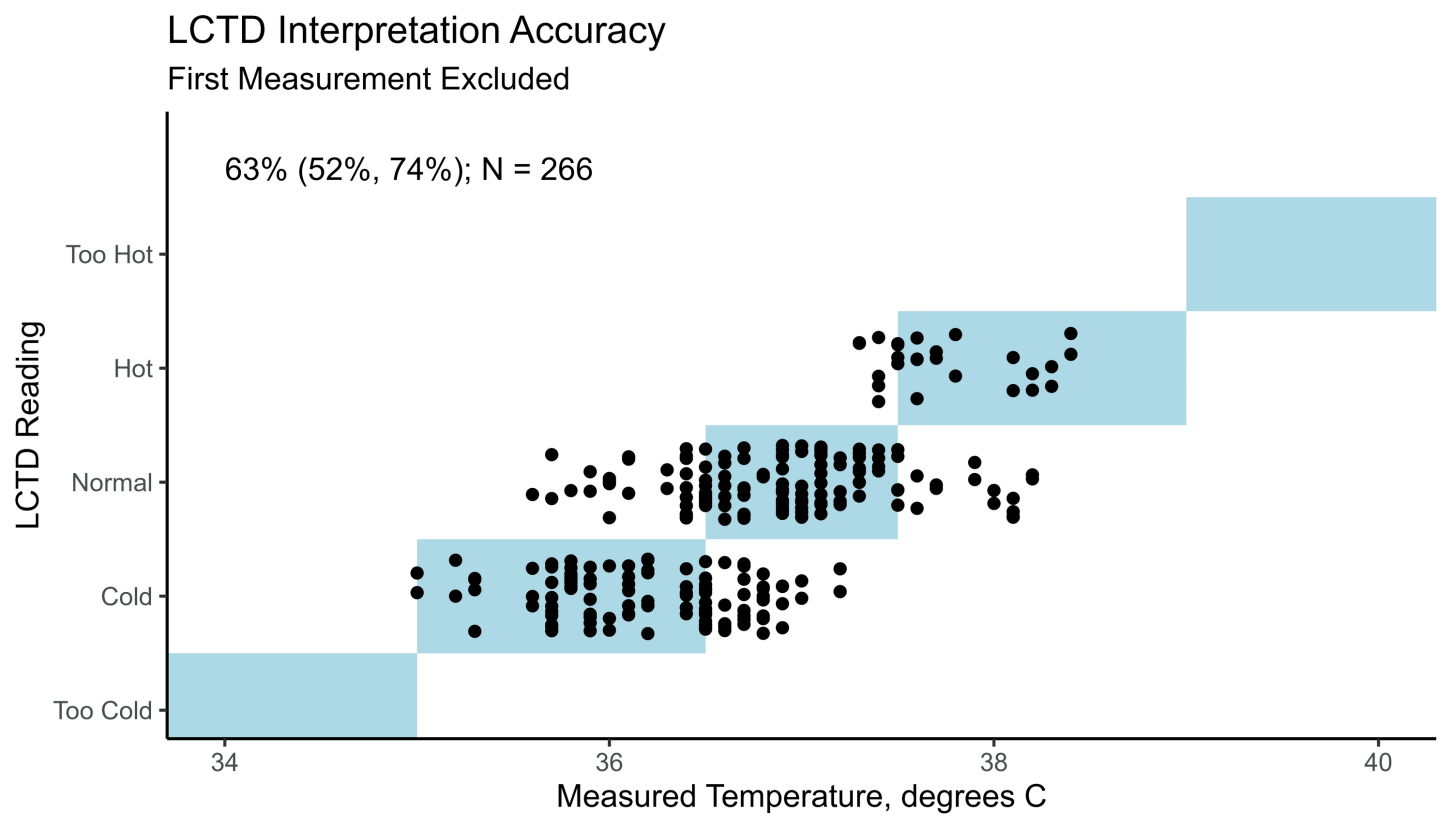
Comparison of LCTD readings by caregivers and research assistants (vertical axis) and measured SoC temperature (horizontal axis) after excluding the first reading per infant Points represent 266 LCTD readings from both research assistants and caregivers corresponding to 133 temperature readings across 40 infants. Points within the light blue boxes on the diagonal indicate observations where the LCTD strip reading implied a temperature range containing the measured SoC temperature. The estimated accuracy (95% confidence interval) is reported in the upper left corner. Note that points have been jittered vertically for visual clarity. LCTD, Liquid Crystal Thermometer Device; SoC, standard of care

Observations identified a latency in the LCTD strip’s ability to accurately register temperature upon initial placement, specifically within the initial five-minute interval. Consequently, a methodological adjustment was implemented to omit the initial reading from each infant's dataset, aiming to mitigate the impact of the LCTD strip’s delayed response in temperature recording. Post-adjustment, the agreement rates (95% CIs) were 71% (44%, 97%) for “cold,” 60% (40%, 79%) for "normal," and 53% (8%, 98%) for "hot" conditions, with an overall concordance rate of 63% (52%, 74%) (Figure 4).

Comparison of accuracy: caregivers vs. research assistants

**Figure 5:**
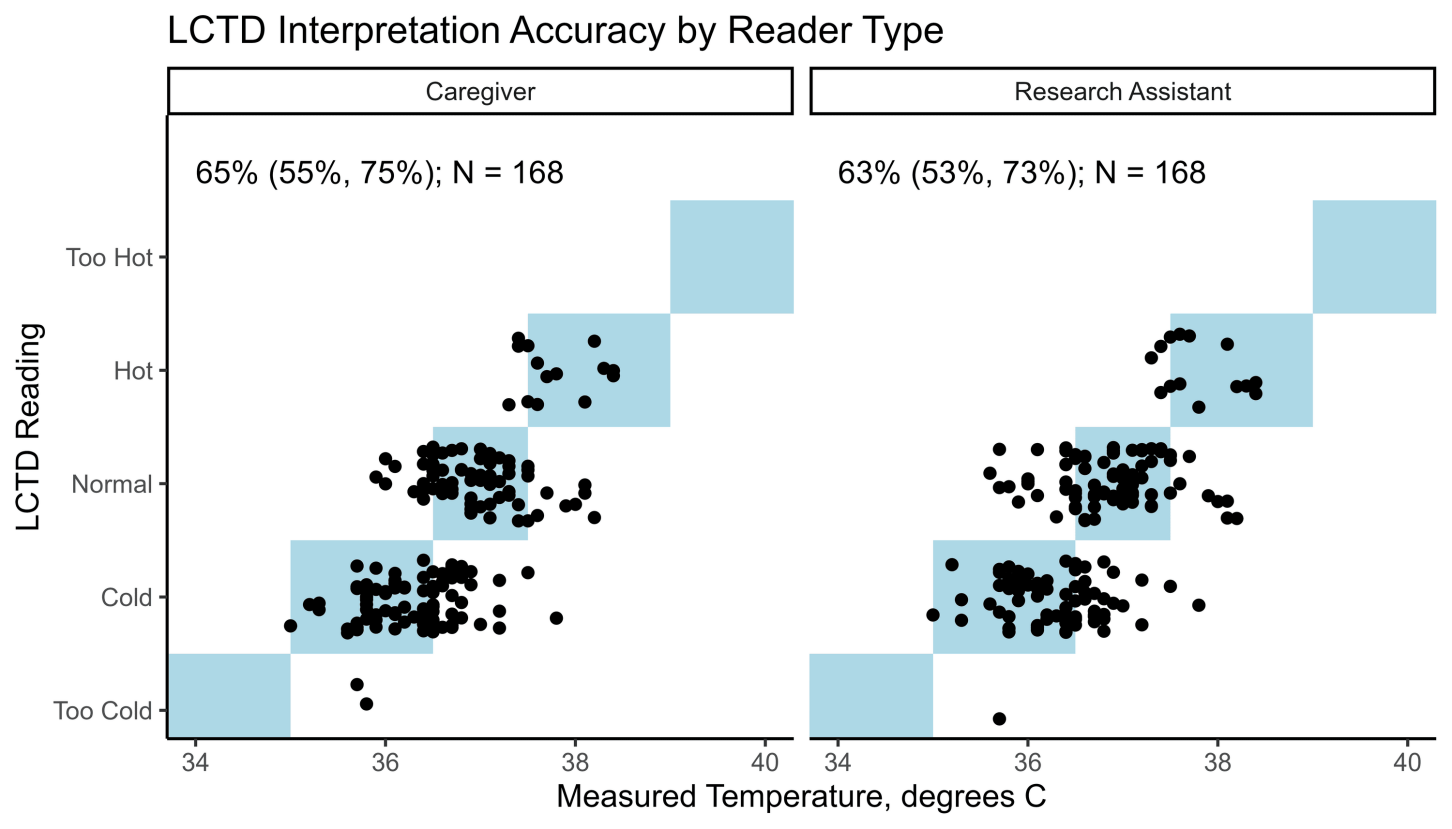
Comparison of LCTD readings by caregivers and research assistants (vertical axis) and measured SoC temperature (horizontal axis) separated by LCTD reader (caregiver vs. research assistant) Points represent 168 LCTD readings from research assistants and 168 LCTD readings from caregivers, corresponding to 168 temperature readings across 40 infants. Points within the light blue boxes on the diagonal indicate observations where the LCTD strip reading implied a temperature range containing the measured SoC temperature. The estimated accuracy (95% confidence interval) is reported in the upper left corner. Note that points have been jittered vertically for visual clarity. LCTD, Liquid Crystal Thermometer Device; SoC, standard of care

Caregiver and research assistant axilla LCTD strip readings were analyzed to ensure agreement with the thermometer-measured SoC temperature. When including the first temperature measurement, the caregivers had an overall accuracy of 65% (55%, 75%), while the research assistants had an overall accuracy of 63% (53%, 73%) (Figure 5).

Blinded photo analysis

**Figure 6:**
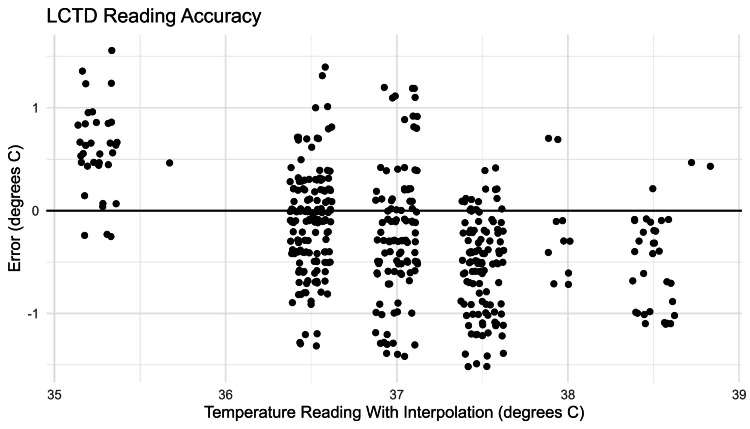
Reading accuracy for the LCTD blinded photo analysis LCTD read temperatures with interpolation are displayed (horizontal axis) with error (measured SoC temperature - LCTD read temperature; vertical axis). Note that points have been jittered horizontally for visual clarity. LCTD, Liquid Crystal Thermometer Device; SoC, standard of care

We summarized the mean and standard deviation (SD) measured SoC temperature within each LCTD strip temperature reading, as well as the average error (defined as the average difference between the measured SoC temperature and the LCTD strip reading, or the midpoint of the two plausible LCTD temperature readings). Across 518 total interpretations, 57 (11%) were labeled as “unclear photo/cannot interpret.” For 163 (31%) interpretations, the research assistants listed two possible readings (e.g., “34 is blue and 36.5 is amber”). In these cases, we interpolated between the two provided temperatures to obtain a single reading temperature for analysis (e.g., 35.25 for the previous example). Across photos that were interpreted, we observed a slight bias for higher temperatures: for readings at or above 37°C, the interpreted temperature tended to be higher than the measured temperature (Table 2, Figure 6).

**Table 2:** Reading accuracy for the LCTD blinded photo analysis All measurements are listed in degrees Celsius. Asterisks (*) denote interpolated readings. The average error was calculated as the average SoC temperature minus the LCTD temperature reading. LCTD, Liquid Crystal Thermometer Device; SD, standard deviation; SoC, standard of care

LCTD temperature reading in ℃	Number of readings	Average SoC temperature in ℃	SD SoC temperature in ℃	Average error in ℃
35.25*	34	35.84	0.44	0.59
35.75*	1	36.20	-	0.45
36.50	154	36.39	0.49	-0.11
37.00*	108	36.77	0.61	-0.23
37.50	110	36.96	0.46	-0.54
38.00*	10	37.82	0.51	-0.18
38.50	31	37.95	0.41	-0.55
38.75*	2	39.20	0.00	0.45

## Discussion

The LCTD strip was evaluated for its accuracy and usability compared to standard digital thermometers. Here, we focused on its potential application in monitoring infants undergoing FSPT. However, we also considered other conditions in infants that require frequent and accurate temperature monitoring, especially in areas in LMICs where HCPs are limited. The findings from this study underscore the challenges and potential of using this modified LCTD strip for temperature monitoring in newborns and young infants, particularly in low-resource settings.

Prior studies have shown the original ThermoSpot™ temperature monitor to be effective in monitoring hypothermia [13]. Pejaver et al. determined that ThermoSpot™ agreed with rectal temperatures 99.04% of the time when monitoring hypothermia (defined as < 35.5°C) [6]. In another study, Kambarami et al. found that ThermoSpot™ was 57% accurate and 19% sensitive in detecting neonatal hypothermia (defined as <36°C), while Green, et al., found a sensitivity of 88% and specificity of 97% when detecting hypothermia (defined as <35°C) in babies born in Indian urban slums [7,8]. The original ThermoSpot™ has been included in several trials to improve neonatal health in rural, low-healthcare-access areas and was also tested in a small pilot study in Nigeria [9,10,12]. However, most research has focused on hypothermia and not on hyperthermia. When NIH PubMed was queried for “liquid crystal thermometer hyperthermia,” only four results populated, spanning back to 1978, highlighting a clear gap in knowledge in monitoring for neonatal hyperthermia. Hence, our team worked with Maternova to modify the original ThermoSpot™ to more accurately monitor for hyperthermia.

The modified LCTD strip initially showed a moderate level of agreement with standard temperature readings, with an overall accuracy of 64%. However, the study identified a latency issue with the device, particularly within the first five minutes of placement. This latency led to a methodological adjustment of excluding the first reading, resulting in a negligible reduction in the LCTD strip's accuracy to 63.5%. This moderate accuracy highlights a critical limitation in the device's current design, suggesting that the LCTD strip may not reliably reflect accurate temperatures.

It is noteworthy that the comparison between caregivers' and research assistants' readings indicated a similar accuracy, suggesting that the usability of the device by non-healthcare professionals might be feasible. However, the device's accuracy issues remain a barrier. At every data collection point, the research assistants returned to the baby and parent/guardian to confirm continued involvement, record the updated LCTD strip reading, and measure SoC temperature. As the caregiver and research assistant responses were recorded at the same time, caregivers could have been influenced to simply reiterate what they saw the research assistant record, or vice versa. The slight increase in caregiver accuracy could have been due to parents being more consistent in their readings because they only observed one baby, while the research assistants rotated between babies.

Study limitations

The LCTD strip has been suggested as a practical and cost-effective temperature monitoring device suitable for low-resource community settings [11]. However, some challenges were encountered during the implementation. The discrepancies in the color gradient proved to be a point of confusion among all readers, with the device sometimes reflecting a blue color and at other times a green color. Additionally, readers sometimes struggled to distinguish whether a window was truly green, or if it was amber or blue, and at times, a single window indicated multiple temperature colors. Sometimes, the device did not reflect any color at all. Another challenge was caregivers losing interest in reading the LCTD midway through the study. This could be related to the challenges in accurately identifying the correct color on the device. Understanding their reasons could inform future study designs.

Study strengths

Our study also has several important strengths, including the use of a diverse, multi-site sample that would have enhanced the generalizability of our findings across different settings and populations. Moreover, the rigor of our iterative process - specifically, our ongoing accuracy checks during preliminary analysis, as well as conducting the study at two diverse sites - allowed us to identify and address issues in real time. This led to meaningful modifications in the placement and interpretation of the LCTD strip, ultimately improving the quality and reliability of the data collection process. These methodological strengths contribute to the robustness and practical relevance of our findings.

## Conclusions

The modified ThermoSpot™ LCTD strip did not correlate well with axillary temperature readings among caregivers or HCPs, with an overall accuracy of 64% and a 95% CI from 54% to 74% (that is, a margin of error of ±10%). Therefore, the true accuracy of the LCTD strip is lower than the 95% we assumed at the start of the study. Additionally, our team encountered methodological challenges in accurately interpreting the LCTD strip, as the color changes were inconsistent and occurred at varying time intervals. Given that the strip is intended for use in LMICs by illiterate caregivers, these difficulties in accurately interpreting the strip pose a problem. Our findings of the LCTD strip’s moderate accuracy and functional inconsistencies indicate it needs further modifications to be effective for neonates undergoing FSPT.

There remains a gap in providing a reliable tool for illiterate caregivers in low-resource areas to assess if their infant is normothermic. While the LCTD shows potential as a low-cost, easy-to-use tool, its current inaccuracies must be addressed. Based on the high accuracy and sensitivity of the original ThermoSpot™, we are optimistic that changes can be made to produce a reliable temperature monitoring tool, especially as it relates to hyperthermia. However, further research and development are needed to improve this LCTD strip’s accuracy and reliability before implementing its widespread use in neonatal care.
